# A Two-Stage Allocation–Transportation Framework with Improved Holistic Swarm Optimization for Port Cargo Transportation Planning

**DOI:** 10.3390/biomimetics11090667

**Published:** 2026-09-17

**Authors:** Cuihua Lu, Yunsheng Li, Lin Yang, Tangying Liu, Yi Wang, Shuxiang Cai

**Affiliations:** 1 The Third School, Naval Aviation University, Yantai 264001, China; llu1978@163.com; 2 School of Electromechanical and Automotive Engineering, Yantai University, Yantai 264005, China; liyunsheng@s.ytu.edu.cn (Y.L.); lty@ytu.edu.cn (T.L.); wy@ytu.edu.cn (Y.W.)

**Keywords:** zone partitioning, improved holistic swarm optimization algorithm, cargo allocation, allocation–transportation two-stage planning method

## Abstract

Due to the high density of warehouse distribution, port cargo is highly susceptible to fire, humidity, and various natural disasters. When these disasters occur, they can result in severe cargo losses. Meanwhile, transportation cost control has long been a prominent research focus in port logistics due to the enormous throughput. To address these problems, this paper proposes a two-stage allocation–transportation framework based on improved holistic swarm optimization. In the allocation stage, this paper designs a warehouse zoning strategy. Based on the distance criterion, a clustering method is employed to group spatially proximate warehouses into the same zone, and distribute the same cargo across different zones. In this way, the same cargo could be prevented from being completely destroyed in a disaster. In the transportation stage, this paper designs an improved holistic swarm optimization algorithm to plan the routes of cargo transportation. In this method, this paper integrates the greedy search algorithm, ant colony optimization, and adaptive swap/reversal operations with the holistic swarm optimization algorithm, which could enhance global search capability and significantly reduce the length of transportation routes. To validate the effectiveness of the proposed algorithm, experiments are conducted on both instances of varying scales and CVRPLIB benchmark instances, and the results are compared with several baseline methods. Specifically, compared with the best-performing baseline ACO, IHSO reduces the optimal route length by up to 2.08% on the self-generated instances and by 5.24% on the CVRPLIB benchmark instances. Moreover, all improvements are statistically significant under the Wilcoxon signed-rank test (*p* < 0.05), confirming that the advantage of IHSO is systematic rather than incidental. The proposed algorithm demonstrates high adaptability in solving port cargo path planning problems, delivering high-quality solutions.

## 1. Introduction

As a critical hub for maritime and overland trans-shipment [1,2], ports have the characteristics of large throughput and centralized cargo storage, which makes them susceptible to natural disasters. In general, the same cargo is stored in a single warehouse for simplicity, high efficiency, and easy implementation. However, if disaster occurs, the cargo may be destroyed entirely, which may disrupt the supply chain and cause the halt of production cycles. This risk can be effectively mitigated by decentralized storage of the same cargo. Nevertheless, excessively dispersed cargo storage can lead to difficulties in plan transportation routes and storage management. Moreover, warehouses are concentrated in ports, which puts forward higher requirements for path planning during the cargo transportation process.

For the port cargo storage allocation problem, the keys to reducing storage risks are rational warehouse zoning and effective cargo allocation. Traditional storage allocation methods mainly include random storage, turnover-rate-based ABC classification storage, and dedicated storage [3]. Among them, ABC classification storage classifies cargo into three categories based on demand frequency. Higher-demand cargo is placed closer to entry and exit points, which effectively reduces picking travel distances [4]. However, these methods focus on slotting optimization with a single warehouse. They do not address cargo allocation across multiple warehouses. In recent years, clustering methods have been widely applied in the zone partitioning stage for routing problems. For example, Yin [5] proposed a cluster-first route-second two-stage method for emergency logistics scheduling. This method first determines the optimal replenishment strategy via continuous approximation. A clustering method then partitions gas stations into zones. Finally, the Christofides algorithm is applied to solve TSP routes within each zone. This method decomposes the complex vehicle routing problem into multiple tractable subproblems, which can reduce the computational difficulty and generate high-quality scheduling solutions. Wang [6] proposed a two-stage clustering–evolutionary framework with adaptive search control. This method uses K-means clustering to partition customers and introduces an adaptive genetic algorithm to optimize the local route within each subregion, which significantly improves the solving efficiency of large-scale VRPTW. Accordingly, in this paper, warehouses are partitioned into two zones based on the distance clustering, which can reduce the risk that the same cargo is completely lost. After zone partitioning, this paper performs cargo allocation based on warehouse capacity and location, thereby simplifying the port cargo transportation problem into a Capacitated Vehicle Routing Problem [7] (CVRP).

CVRP is an important research area in path planning problems. Its objective is to plan transportation routes subject to vehicle capacity constraints, so as to minimize the total travel distance. There are three main types of CVRP solution: exact algorithms [8], deep learning algorithms [9], and swarm intelligence algorithms [10]. Exact algorithms are a kind of classic algorithm for solving the CVRP, which refers to a type of algorithm that can calculate the shortest path. It is a classic algorithm for solving the CVRP. Exact algorithms, such as branch and bound method [11], column generation method [12], and branch and cut method [13], are able to find the globally optimal solution. However, as the problem scale grows, the computational complexity of the algorithm grows exponentially. This makes it difficult to obtain the optimal solution within an acceptable time frame [14,15,16]. Deep learning algorithms are an end-to-end path planning method, which can directly plan a path by calculating the input representations. In routing problems with a large number of nodes, deep learning algorithms such as graph attention networks [17], graph convolutional networks [18], and deep Q-Networks [19] can rapidly obtain solutions. However, training of deep learning algorithms requires large datasets. When the dataset is small, the model generalizes poorly and this often fails to meet practical requirements. Moreover, deep learning methods impose high hardware requirements during both training and inference [20,21,22], which significantly constrains their widespread adoption and deployment.

Swarm intelligence algorithms are a classical family of routing methods which seek optimal solutions through information exchange and cooperation among individuals. These methods have low computational complexity, which can generate high-quality solutions within an acceptable time range. Typical swarm intelligence methods such as the harris hawks optimization algorithm [23], Ant Colony Optimization (ACO) [24], gray wolf optimization algorithm [25], and firefly algorithm [26], can solve complex problems and have high flexibility. However, the parameter sensitivity of these methods is relatively high, which leads to results falling into local optima. To solve this problem, researchers have proposed hybrid algorithms that integrate multiple methods to enhance their robustness. Kao [27] proposed a hybrid algorithm based on ACO and particle swarm optimization. This method allows each ant to record both its personal best and the global best solution, but only allows elite ants to update pheromones. Meanwhile, the method introduces pheromone disturbance and memory reset mechanisms, which effectively accelerate the convergence process and prevent premature convergence to local optima. Pham [28] proposed a global optimization framework which integrates the whale optimization algorithm with the gray wolf optimizer. This method incorporates opposition-based learning and mutation strategies to balance global exploration and local exploitation. This prevents the planning process from becoming trapped in local optima. In addition, it achieves time–cost bi-objective optimization in cement distribution tasks. Zhou [29] proposed a hybrid bat algorithm incorporating a path relinking strategy, which introduces greedy randomized adaptive search and single-point local search to prevent the algorithm from falling into local optima. Moreover, the method enables rapid convergence to high-quality CVRP solutions. Therefore, the improvement of swarm intelligence algorithms remains a research hotspot in this field.

In recent years, scholars have continuously proposed new optimization algorithms [30,31,32] to enhance solution performance. Among them, Holistic Swarm Optimization [33] (HSO) has attracted considerable attention due to its outstanding performance in continuous function optimization and engineering design. To date, research on HSO has not been applied in the field of discrete combinatorial optimization, such as vehicle routing. To enable HSO to better solve discrete problems, this study draws inspiration from other well-established optimization algorithms. Among these algorithms, ACO is a classic biomimetic algorithm, which simulates the pheromone-mediated foraging behavior of ants to search for optimal solutions. This algorithm encodes the solutions as sequences of discrete nodes, making it naturally suitable for solving discrete problems. HSO is essentially a continuous-space optimizer with powerful global search and convergence capabilities. Integrating ACO into HSO can effectively enhance its ability to solve discrete problems such as vehicle routing. To further strengthen this performance, the greedy search algorithm is also introduced to construct a high-quality initial population, while the swap and reversal operations are adopted to generate new offspring, so as to improve the ability to escape local optima and eliminate route crossings. Therefore, this paper proposes a novel Improved Holistic Swarm Optimization (IHSO) method. The method effectively integrates the greedy search algorithm [34], ACO [35], adaptive swap/reversal operations [36,37], and adaptive simulated annealing strategy. It improves initial solution construction, enhances the ability to escape local optima, and increases global convergence speed. These improvements enable efficient CVRP solving with superior results.

The main contributions of this paper include the following:

1. To balance storage safety and transportation cost for the cargo in ports, this paper proposes a two-stage allocation–transportation planning method. In this method, warehouses are partitioned into zones using a clustering approach and a route planning strategy is designed, which achieves a balance between storage safety and transportation cost.
2. An Improved Holistic Swarm Optimization algorithm is proposed which effectively integrates the greedy search algorithm, ACO, adaptive swap/reversal operations, and adaptive simulated annealing strategy. It achieves high convergence speed while reducing the total vehicle travel distance.
3. In multiple sets of experiments on the port cargo transportation problem, IHSO outperforms the comparison algorithms in both solution quality and convergence speed.

The remainder of this paper is organized as follows: Section 2 provides a detailed description of the problem model and its mathematical formulation; Section 3 introduces the optimization algorithms involved, including HSO, ACO, the greedy search algorithm, and adaptive swap/reversal operations, and elaborates on and analyzes the proposed IHSO; Section 4 demonstrates the application of IHSO to the port cargo planning problem; and finally, the paper concludes with a summary of the findings.

## 2. Port Cargo Allocation and Transportation Path Planning Problem

The port cargo allocation and transportation path planning problem is a problem that includes allocation stage and transportation stage. In allocation stage, cargo is allocated to warehouses based on capacity and distance. In the transportation stage, vehicles then deliver the cargo and return to the starting point, such that the total travel distance is minimized. From the perspective of management convenience and transportation efficiency, traditional approaches often store the same cargo centrally in a single warehouse. However, centralized storage may pose several risks. In the event of sudden disasters such as fire, flooding, or earthquakes, centrally stored cargo is highly vulnerable to complete destruction. This may disrupt the supply chain and cause severe economic losses. To address the above problems, this paper proposes a two-stage allocation–transportation framework based on improved holistic swarm optimization. In the allocation stage, first, this paper employs a distance-based clustering method to partition warehouses into two zones, and randomly selects one warehouse from each zone. Then, cargo is allocated to selected warehouses in proportion to their remaining capacity. This design could fully utilize warehouse space and effectively reduce the risk that the same cargo is destroyed entirely. In the transportation stage, the paper proposes an IHSO, which plans vehicle routes according to warehouse locations and total cargo demand. This effectively reduces the total vehicle travel distance, saves transportation time, and improves transportation efficiency.

### 2.1. Port Cargo Allocation Rules

#### 2.1.1. Zone Partitioning

This paper adopts a Euclidean distance-based clustering method to partition warehouse zones. Let the warehouse be set to I, where $I=\left\{0,1,2,\ldots ,n\right\}$. By constructing two seed points, warehouses are divided into two zones. First, the first warehouse is selected as the initial reference point, and the warehouse farthest from it is taken as the first seed point. This paper denotes them as *p* and $s_{1}$, respectively. Second, the warehouse furthest from $s_{1}$ is taken as the second seed point $s_{2}$. Finally, the distance of each warehouse to $s_{1}$ and $s_{2}$ is calculated, and is assigned to the nearer zone.

For warehouse i, the mathematical expression for its zonal affiliation is

(1) $$I_{i}\in \left\{\begin{array}{c} A,d_{is_{1}}\leq d_{is_{2}}\\ B,otherwise \end{array}\right.$$

where $d_{is_{1}}$ and $d_{is_{2}}$ denote the distances from i to $s_{1}$ and $s_{2}$, respectively. *A* and *B* denote two warehouse area sets.

#### 2.1.2. Cargo Allocation

To improve warehouse utilization, this study designs an allocation rule after zone partitioning. Concretely, the warehouse and cargo sets are designated as I and Z, respectively, where $I=\left\{0,1,2,\ldots ,n\right\}$ and $Z=\left\{0,1,2,\ldots ,n\right\}$. Warehouse i has capacity $U_{i}$ and its remaining capacity is $R_{i}$. Cargo z has quantity $Q_{z}$ and remaining quantity $Q_{z}^{\prime }$. Thus, $R_{i}$ and $Q_{z}^{\prime }$ are initialized to $U_{i}$ and $Q_{z}$. $X=\left[x_{iz}\right]$ is defined as the allocation matrix, where $x_{iz}$ denotes the quantity of cargo z assigned to warehouse i. All entries are initialized to zero. $C_{A}=\left\{i\in A|R_{iA}>0\right\}$ and $C_{B}=\left\{i\in B|R_{iB}>0\right\}$ denote the sets of warehouses with remaining capacity in zones A and B, respectively.

For cargo z, when $Q_{z}^{\prime }>0$ and there exists a warehouse with $R_{i}>0$, the allocation is performed according to the following rules:

1. Warehouse selection: If both $C_{A}$ and $C_{B}$ are non-empty, this paper randomly selects a warehouse from each area to participate in allocation. This ensures that the same cargo is stored dispersedly across different zones. In addition, if only one zone has available warehouses, at most two warehouses are randomly selected from that zone.
2. Allocation quantity calculation: The total remaining capacity of the selected warehouses is
  (2) $$R_{tatal}=R_{a}+R_{b}$$
  where $R_{a}$ and $R_{b}$ denote the remaining capacity of the selected warehouses.

The allocation quantity should take the smaller value between the total remaining warehouses’ capacity and the remaining cargo quantity, so as to prevent the allocated cargo quantity from exceeding the total remaining warehouse capacity. The formulation is

(3) $$t=\min\left\{Q_{z}^{\prime },R_{tatal}\right\}$$

3. Cargo allocation: If only one warehouse is selected, that warehouse receives the entire allocation quantity t. If two warehouses are selected, the cargo is allocated in proportion to their remaining capacities. Proportional distribution ensures balanced cargo distribution. It avoids the situation in which the larger warehouses are underutilized while the smaller one is full. This improves the overall warehouse utilization rate. The allocation quantities for the two warehouses are
  (4) $$A_{a}=t\cdot \frac{R_{a}}{R_{tatal}}$$
  (5) $$A_{b}=t-\Delta R_{a}$$
  where $A_{a}$ and $A_{b}$ denote the quantity of cargo allocated to each selected warehouse, respectively.
4. Allocation matrix, warehouse and cargo status updating: After allocation, the allocation matrix $X=\left[x_{iz}\right]$ is updated as follows:
  (6) $$x_{iz}\leftarrow x_{iz}+A_{i}$$
  where $A_{i}$ denotes the quantity of cargo received by warehouse i.

The remaining capacity of each warehouse and remaining capacity of each cargo type are updated as:

(7) $$R_{i}\leftarrow R_{i}-\Delta R_{i}$$

(8) $$Q_{z}^{\prime }\leftarrow Q_{z}^{\prime }-t$$

5. Termination condition: The allocation process is terminated when all warehouses are full or all cargo has been allocated. Finally, the output is the allocation matrix $X=\left[x_{iz}\right]$.

### 2.2. Mathematical Model of the Port Cargo Transportation Problem

After cargo allocation, the port cargo transportation problem is reduced to a CVRP. Define a directed graph $G=\left(V,A\right)$, where V is the node set, V={0,1,2,…,n}. Node 0 represents the port and N={1,2,…,n} is the warehouse set. Warehouse i has demand $q_{i}$. A is the arc set, and the straight linear distance of arc $\left(i,\;j\right)$ is $c_{ij}$. Let K denote the set of delivery vehicles with uniform capacity C, K={1,2,…,k}. A feasible route in the directed graph G must start and end at the port. Furthermore, ${\left(i\right)}^{+}$ denotes the set of arcs departing from node i, and ${\left(j\right)}_{-}$ denotes the set of arcs arriving at node j.

The mathematical expression of the minimum total distance of the truck route is defined as follows:

(9) $$\min\underset{k\in K\left(i,j\right)\in A}{\sum }c_{ij}x_{ijk}$$

where $c_{ij}$ denotes the distance between warehouses i and j, and $x_{ijk}$ denotes whether truck k departs from warehouse i and arrives at warehouse j. If so, $x_{ijk}$ = 1; otherwise, $x_{ijk}$ = 0. In summary, the mathematical model of the port cargo transportation problem is as follows:

(10) $$\min\underset{k\in K\left(i,j\right)\in A}{\sum }c_{ij}x_{ijk}$$

(11) $$\sum_{k\in K,j\in {\left(i\right)}^{+}}x_{ijk}=1\;\forall i\in N$$

(12) $$\sum_{j\in {\left(0\right)}^{+}}x_{0jk}=1\;\forall k\in K$$

(13) $$\sum_{i\in {\left(j\right)}_{-}}x_{ijk}-\sum_{i\in {\left(j\right)}^{+}}x_{ijk}=0\;\forall k\in K,\forall j\in N$$

(14) $$\sum_{i\in {\left(n+1\right)}_{-}}x_{i,n+1,k}=1\;\forall k\in K$$

(15) $$\sum_{i\in N}q_{i}\sum_{j\in {\left(i\right)}^{+}}x_{ijk}\leq C\;\forall k\in K$$

(16) $$\sum_{i\in S}\sum_{j\in S}x_{ijk}\leq |S|-1\;\forall S\subseteq N,|S|\geq 2,\forall k\in K$$

(17) $$x_{ijk}\in \{0,1\}\;\forall k\in K,\forall (i,j)\in A$$

Equation (10) minimizes the total travel distance of all trucks. Equation (11) ensures that each warehouse is served by exactly one route. Equation (12) ensures each truck departs from the port once. Equation (13) maintains flow conservation at each warehouse j. For each truck k, the inflow equals the outflow. Equation (14) requires each truck k to return to the port after replenishment. Equation (15) ensures that the load capacity of truck k does not exceed the maximum capacity after replenishment. Equation (16) ensures that all subtours pass through the port, where set S is a subset of set N. Equation (17) denotes that if truck k departs from warehouse i to warehouse j, $x_{ijk}$ = 1; otherwise, $x_{ijk}$ = 0.

## 3. Improved Holistic Swarm Optimization

### 3.1. Holistic Swarm Optimization

HSO [33] is a metaphor-less swarm intelligence optimization algorithm which leverages the entire population’s information to enhance the search process. It enhances the ability to explore unknown search spaces and exploit information from superior individuals through root-mean-squared fitness-based displacement coefficients and adaptive mechanisms. Thus, it can effectively solve multimodal optimization problems in complex environments.

The specific implementation of HSO is as follows:

1. Initialization: Each search individual is randomly generated at an initial position $x_{i}$. Then, this paper sets the maximum number of iterations to be $iter_{max}$, and the initial mutation rate and step to be $\lambda_{\mathrm{init}}$ and $\delta_{\mathrm{init}}$. Finally, the final mutation rate, step, the initial temperature and the cooling rate are set to be $\lambda_{\mathrm{final}}$, $\delta_{\mathrm{final}}$, $T_{initial}$ and CR, respectively.
2. Fitness evaluation: Evaluate the fitness $f\left(x_{i}\right)$ of each search individual.
3. Adaptive simulated annealing strategy: When the new fitness value outperforms the old one, the new value directly replaces the old one. When the new value is inferior, the algorithm still accepts it if the probability P is greater than a uniformly distributed random number. The probability P is formulated as
  (18) $$P=exp\left(-\frac{\Delta f}{T_{initial}CR^{iter}}\right)$$
  where Δf denotes the difference between the new and old fitness values, and iter denotes the current iteration number.
4. Update the best solution and compute the displacement coefficient: the root mean square of all individual fitness values is calculated, denoted as ${\mathrm{RMS}}_{f}$. Its mathematical expression is
  (19) $${\mathrm{RMS}}_{f}=\sqrt{\frac{1}{n}\sum_{i=1}^{n}f{\left(x_{i}\right)}^{2}}\;$$

The mathematical expression for the displacement coefficient $c_{i}$ is

(20) $$c_{i}=\frac{\mathrm{sign}\left(d_{i}\right)\cdot \left|d_{i}\right|}{\sum_{j=1}^{n}\left|d_{j}\right|}$$

where $d_{i}$ denotes the difference between the fitness value of individual $x_{i}$ and ${\mathrm{RMS}}_{f}$ values.

5. Population individual update: Each individual $x_{i}$ is updated to approach high-quality individuals and moves away from inferior individuals. The population individual update rule is
  (21) $$x_{i}^{\prime }=x_{i}+\alpha \overset{n}{\underset{j=1}{\sum }}r_{ij}\cdot c_{j}\cdot \left(x_{j}-x_{i}\right)$$
  where α is a constant parameter and $r_{ij}$ is a random value; $c_{j}$ is the displacement coefficient.
6. Adaptive mutation: The mutation rate and step size gradually change linearly from $\lambda_{\mathrm{init}}$ and $\delta_{\mathrm{init}}$ to $\lambda_{\mathrm{final}}$ and $\delta_{\mathrm{final}}$ as iterations increase. If adaptive mutation is triggered, a random perturbation based on a normal distribution is added to the individual $x_{i}$. This produces a new individual $x_{i}^{\prime }$.
7. Termination check: Repeat steps 2 through 7 until the maximum number of iterations is reached.

### 3.2. Ant Colony Optimization

ACO [35] is a classical swarm intelligence algorithm inspired by ant foraging behavior. During foraging, ants preferentially select paths with higher pheromone concentrations. Shorter paths accumulate more pheromones, and these paths are more likely to be chosen. As iterations proceed, the pheromone concentration on shorter paths continuously accumulates. This enables the algorithm to progressively find the shortest path. For vehicle routing problems, ACO generates visit sequences through pheromone-guided route construction. It only allows some top-performing ants to update the pheromone, which strengthens the ability to find better routes. Accordingly, IHSO incorporates the elitist pheromone update and heuristic guidance from ACO to enhance its global convergence capability.

### 3.3. Greedy Search Algorithm

The greedy search algorithm [34] is a simple heuristic method. Its core idea is to construct a feasible solution of reasonably high quality by selecting the locally optimal decision at each step. For vehicle routing problems, the greedy algorithm starts at the depot, always moves to the nearest unvisited node, and returns to the depot after visiting all nodes. To obtain a high-quality initial population, population initialization of IHSO combines the greedy search algorithm and randomly generated solutions. The greedy solution effectively improves the overall population quality, while the random solutions help increase the diversity of the initial population.

### 3.4. Swap Operation and Reversal Operation

Swap operation [36] and reversal operation [37] generate new offspring by recombining parental genes, which effectively enhance the ability to escape local optima and further expand the search space. The swap operation is simple and efficient, and helps strengthen the ability to escape local optima. The reversal operation can effectively eliminate route crossings, shorten route distances, and enhance the local optimization capability. Therefore, this paper introduces the swap operation and the reversal operation to replace the mutation operation in the adaptive mutation mechanism.

**Figure 1:**
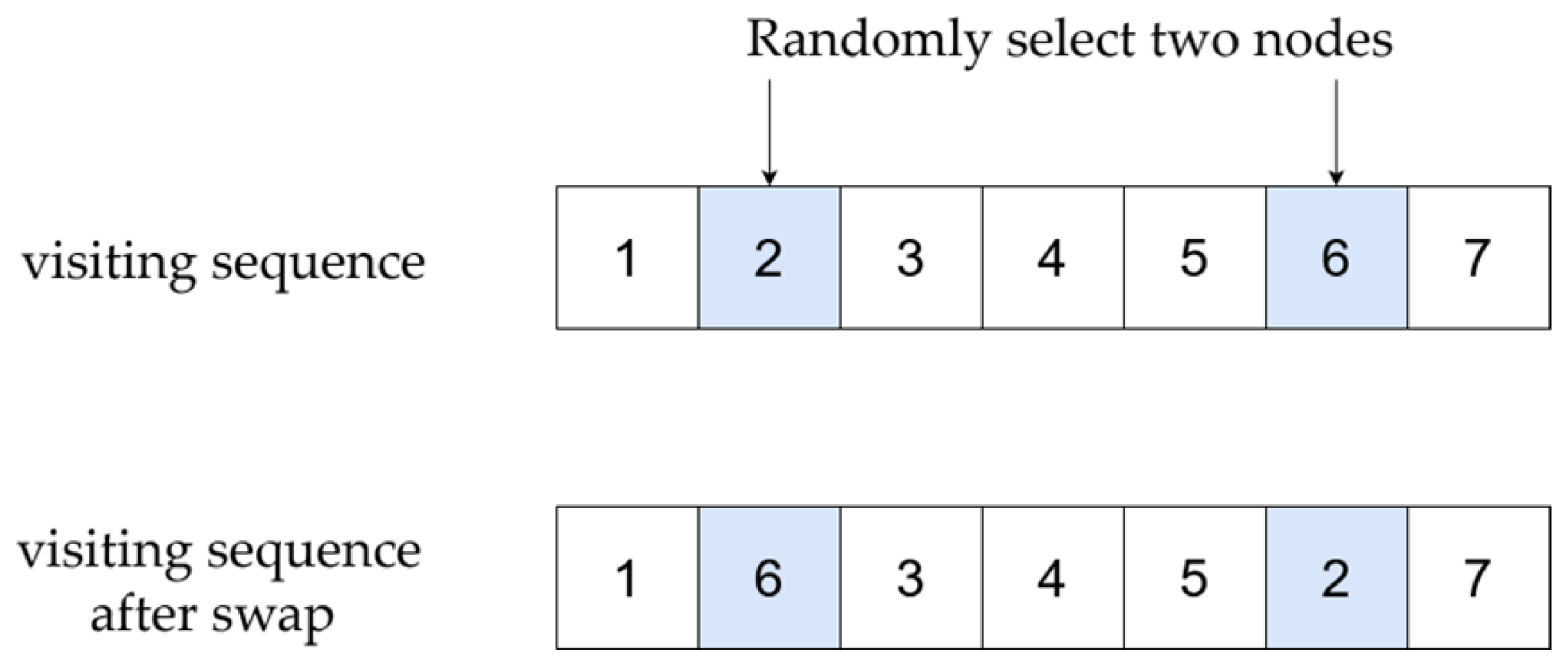
Illustration of the swap operation.

When the swap operation is executed, it randomly selects two nodes in the visit sequence and then swaps their positions, while all other positions remain unchanged. The operation is shown in Figure 1.

**Figure 2:**
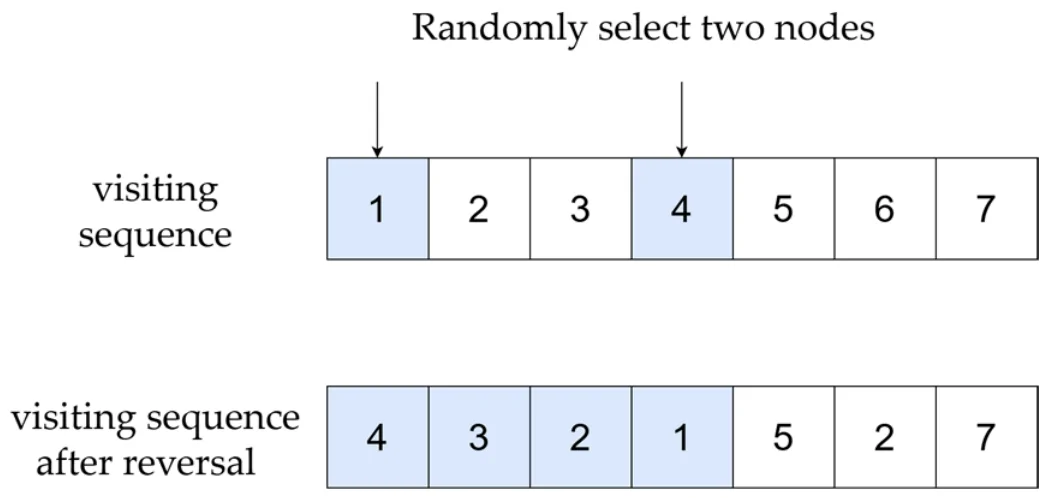
Illustration of the reversal operation.

When the reversal operation is executed, it randomly selects two nodes in the visit sequence and reverses the order of all nodes between them, while all other positions remain unchanged. The operation is shown in Figure 2.

### 3.5. The Proposed Improved Holistic Swarm Optimization

**Figure 3:**
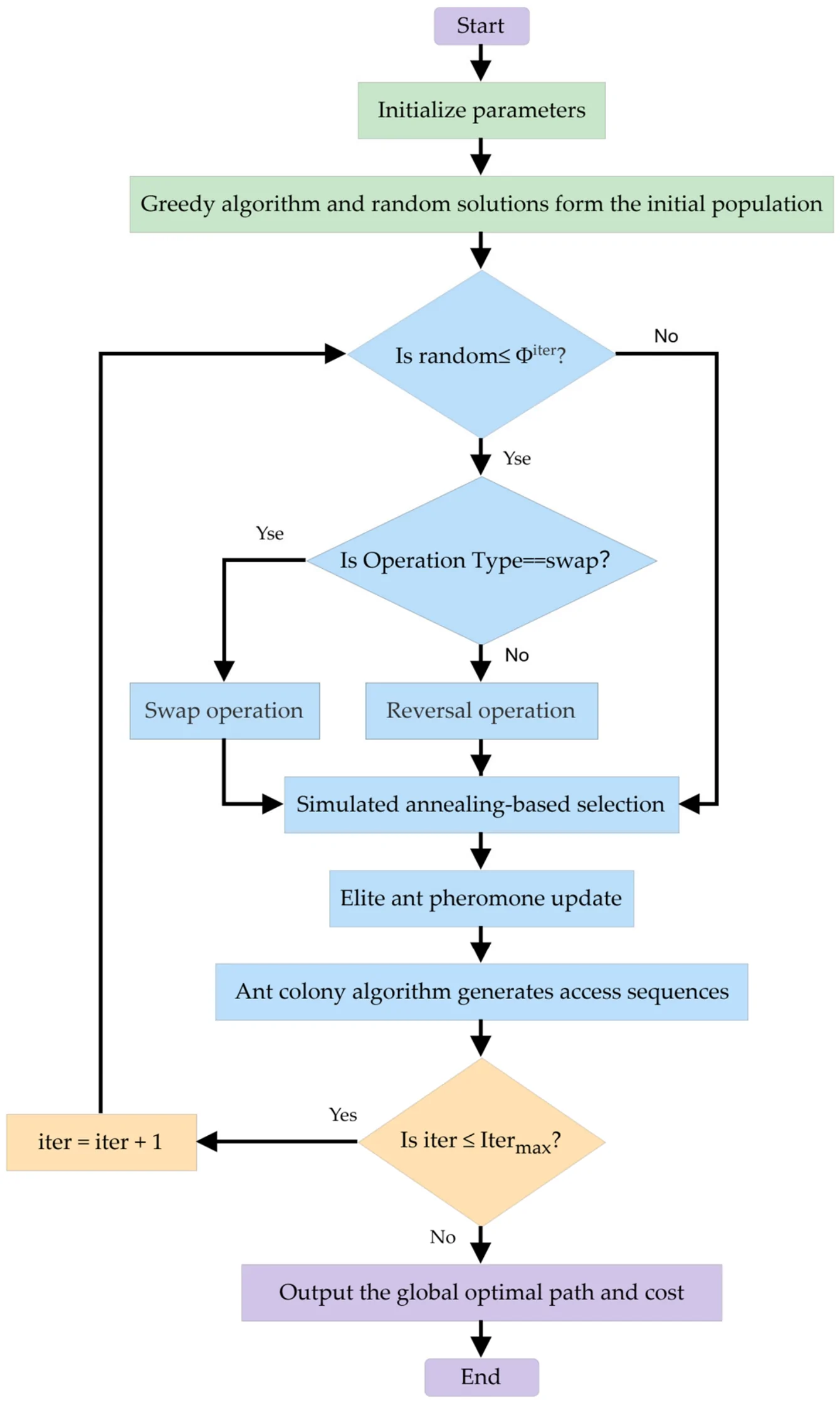
Flow chart of the improved holistic swarm optimization.

To further improve solution quality, this paper improves HSO by effectively integrating the greedy search algorithm, adaptive swap/reversal operations, and ant colony optimization. This could further balance the ability to explore unknown search spaces and exploit known high-quality information. In order to solve the port cargo transportation problem, this paper employs the swap/reversal operations and ACO to replace the mutation operation and the population position update, respectively. That avoids entrapment in local optima and accelerates global convergence. The flow chart of IHSO is illustrated in Figure 3.

The detailed working procedure of IHSO is as follows:

1. Initialization: The initial population is composed of individuals generated by the greedy search algorithm and randomly generated method. In this paper, the maximum number of iterations, the initial operation rate and final operation are set to be $iter_{max}$, $\Phi_{\mathrm{init}}$ and $\Phi_{\mathrm{final}}$, and the initial temperature and cooling rate are set to be T0 and ζ. Meanwhile, the pheromone importance factor, the heuristic importance factor, the pheromone evaporation factor and the pheromone increment constant are set to be α, β, ρ and Q, respectively.
2. Adaptive swap/reversal operation: If the probability $\Phi^{iter}$ is greater than the random number drawn from a uniform distribution, this algorithm randomly selects the swap operation and reversal operation with equal probability and executes the chosen operation. The mathematical expression for the probability $\Phi^{iter}$ is:
  (22) $$\Phi^{iter}=\Phi_{\mathrm{init}}-iter\times \left(\frac{\Phi_{\mathrm{init}}-\Phi_{\mathrm{final}}}{iter_{max}}\right)$$
3. Adaptive simulated annealing strategy: If the new solution is better than the old solution, the new solution directly replaces the old solution; if the new solution is worse than the old solution, the old solution is accepted with probability $P_{SA}$. The mathematical expression of the probability $P_{SA}$ is
  (23) $$P_{SA}=exp\left(-\frac{C_{new}-C_{old}}{T0\cdot \zeta^{iter}}\right)$$
  where $C_{new}$ denotes the new solution and $C_{old}$ denotes the old solution.
4. Elitist ant pheromone, the minimum path cost, and optimal transportation path update:
  (24) $$\tau_{ij}(t+1)=(1-\rho )\tau_{ij}(t)+\Delta \tau_{ij}(t)$$
  (25) $$\Delta \tau_{ij}(t)=\sum_{k\in \varepsilon }^{n}\Delta \tau_{ij}^{k}(t)$$
  (26) $$\Delta \tau_{ij}^{k}=\left\{\begin{array}{ll} \frac{Q}{L_{k}}, & if\;arc\;(i,\;j)\;is\;used\;by\;antk\\ 0, & otherwise \end{array}\right.$$
  where $L_{k}$ denotes the total length of the route traversed by ant k, and ε denotes the set of elite ants.
5. Construction of access sequence based on ACO: Ants depart from the port and progressively select the next node based on pheromone concentration and heuristic information, until all warehouses have been visited. The probability of ant k moving from node i to node j is
  (27) $$P_{ij}^{k}\left(t\right)=\left\{\begin{array}{ll} \frac{{\left[\tau_{ij}\left(t\right)\right]}^{\alpha }{\left[\eta_{ij}\left(t\right)\right]}^{\beta }}{\sum_{s\epsilon allowed_{k}}{\left[\tau_{is}\left(t\right)\right]}^{\alpha }{\left[\eta_{is}\left(t\right)\right]}^{\beta }}, & j\epsilon allowed_{k}\\ 0, & otherwise \end{array}\right.$$
  where $allowed_{k}$ denotes the set of nodes currently selectable by ant k, $\tau_{ij}\left(t\right)$ denotes the pheromone concentration from node i to node j at time t, and $\eta_{ij}\left(t\right)$ denotes the heuristic information from node i to node j at time t. The mathematical expression for $\eta_{ij}\left(t\right)$ is
  (28) $$\eta_{ij}\left(t\right)=\frac{1}{d_{ij}}$$
  where $d_{ij}$ denotes the distance from node i to node j.
6. Termination check: Repeat steps 2 through 6 until the maximum number of iterations is reached.

The algorithm for IHSO is presented in Algorithm 1.
**Algorithm 1:** Improved Holistic Swarm Optimization**Input:**$\;\mathrm{Warehouse}\;\mathrm{node}\;\mathrm{set},\;\mathrm{vehicle}\;\mathrm{capacity}\;\mathrm{constraint},\;\mathrm{demand}\;\mathrm{of}\;\mathrm{each}\;\mathrm{node},\;\mathrm{and}\;\mathrm{algorithm}\;\mathrm{parameters}\;(iter_{max}$ $\Phi_{\mathrm{final}}$$,\;\Phi_{\mathrm{init}}$, ζ, T0, α, β, ρ and Q)**Output**: Global best solution and corresponding routes**Phase 1**: Population InitializationInitialize ACO parameters, simulated annealing parameters, adaptive mutation parameters and maximum number of iterations $iter_{max}$Generate $P_{rand}$ (m is the number of ants)Generate one solution $P_{1}$ using the greedy search algorithmCombine $P_{rand}$ and $P_{1}$ jointly form the initial population **Phase 2**: Route Optimization5.For iter$\;=\;1\;\mathrm{to}\;iter_{max}$ do6.Perform adaptive swap/reversal operation with probability $\Phi^{iter}$7.Apply the adaptive simulated annealing strategy to accept or reject new solution8. Update the elitist ant pheromone and the global best solution9. Construct visit sequences using ACO10.End11.Return the global best solution and route

## 4. Application of the Improved Holistic Swarm Optimization Algorithm to the Port Cargo Transportation Problem

To evaluate the performance of the proposed algorithm, this paper compares IHSO with ACO [38], GA [39], PSO [40], HSO, and SA [41] under identical conditions. All algorithms run on a hardware platform with an Intel Core i7-11800H processor, an NVIDIA GeForce RTX 3050 Ti Laptop GPU, 16 GB RAM, Python 3.14 and Windows 10 64-bit operating system. The parameters of the IHSO are shown in Table 1.

This paper constructs six instances of different sizes. The number of warehouses and the number of cargo types are 100, 200, 300, 400, 500, and 600, respectively. Warehouses that receive no cargo after the allocation stage are excluded from route planning. Consequently, the numbers of warehouses actually participating in the route planning are 95, 185, 271, 374, 463, and 559, respectively. In this study, two integers—one between 100 and 150 and the other between 80 and 100—are randomly generated for warehouse capacity and cargo quantity, respectively. The port coordinates are fixed at (400, 500), and the truck capacity is uniformly set to 200. IHSO, HSO, ACO, GA, PSO, and SA are employed to solve the above six instances, respectively. In addition, to evaluate performance differences between IHSO and the compared algorithms, this paper uses the Wilcoxon signed-rank test in six instances. Finally, to improve the reproducibility and external validity of the results, this paper sets two standard CVRP benchmark instances from CVRPLIB.

**Figure 4:**
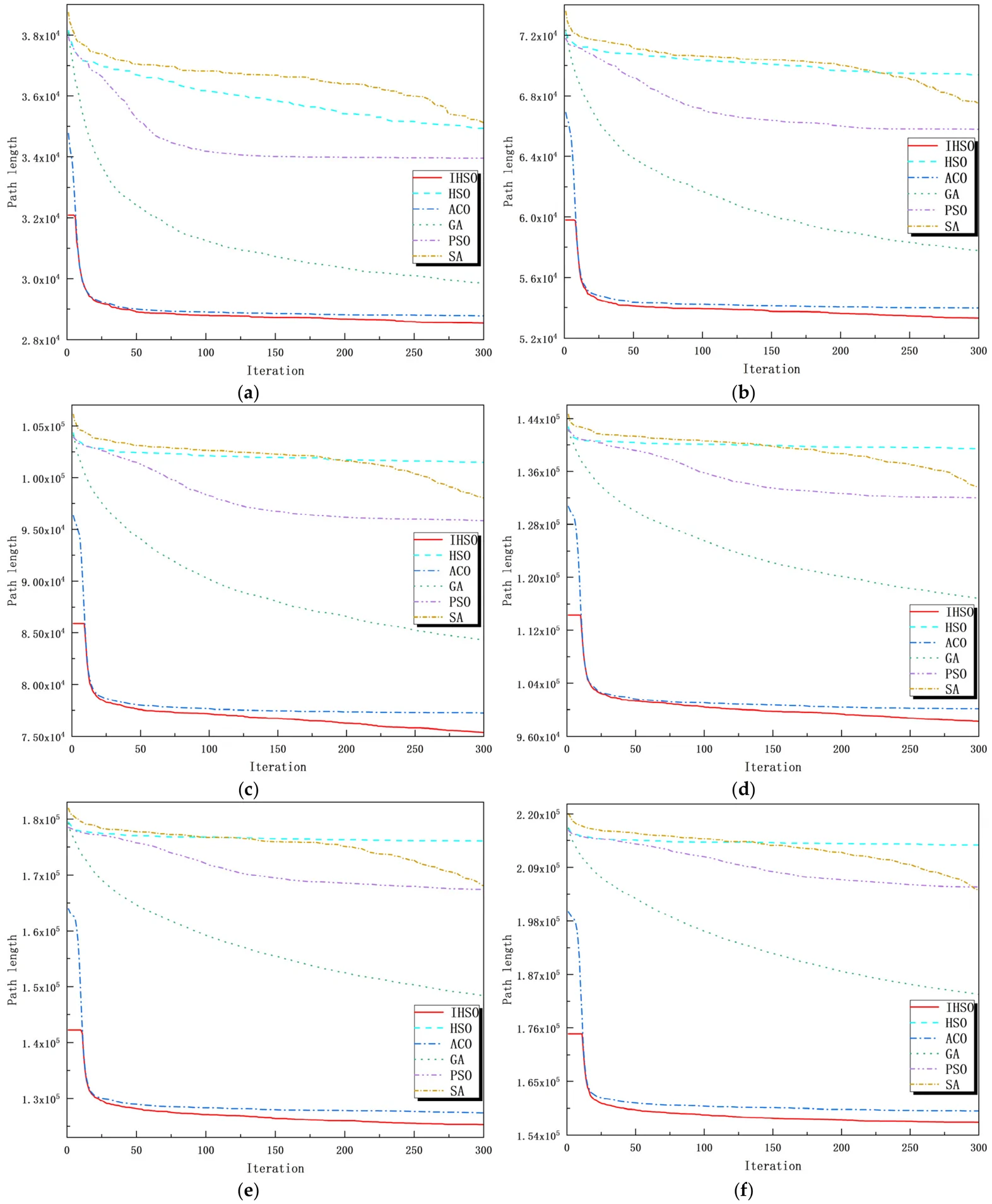
The convergence curves averaged over 20 independent runs for each algorithm: (**a**) 95 warehouses; (**b**) 185 warehouses; (**c**) 271 warehouses; (**d**) 374 warehouses; (**e**) 463 warehouses; (**f**) 559 warehouses.

**Figure 5:**
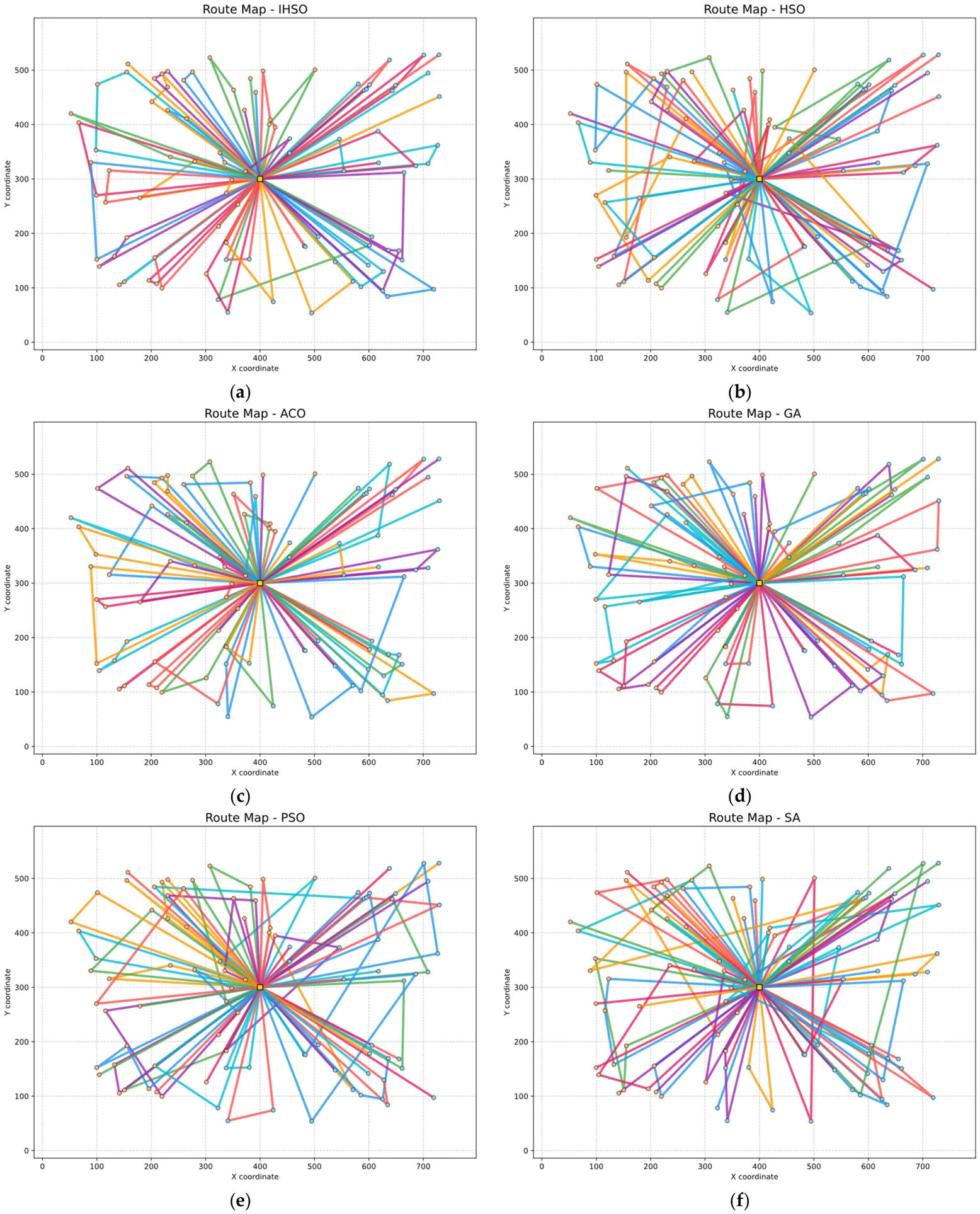
Optimal routes obtained by six algorithms for 95 warehouses. (**a**) IHSO; (**b**) HSO; (**c**) ACO; (**d**) GA; (**e**) PSO; (**f**). SA.

**Figure 6:**
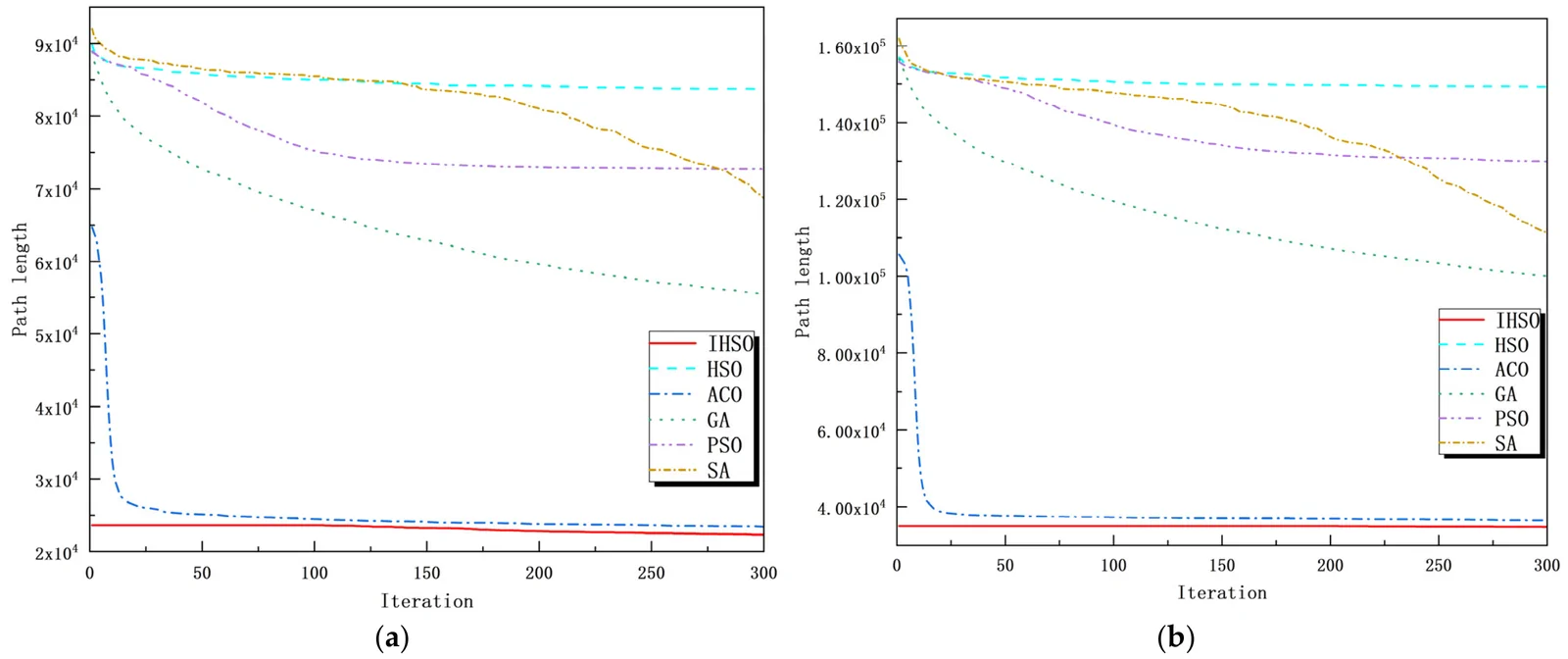
The convergence curves averaged over 20 independent runs for each algorithm. (**a**) X-n204-k19; (**b**) X-n308-k13.

The population size of all algorithms and the maximum number of iterations are set to 50 and 300, respectively. Among them, the elite ant population size of ACO and IHSO is 25. Each algorithm is independently executed 20 times. This paper records the optimal value of each run and simultaneously computes the Best (best value), Ave (mean of the best values), and Std (standard deviation of the best values) over the 20 runs. The data are presented in detail in Table 2. Figure 4 illustrates the average convergence curves of the IHSO, HSO, ACO, GA, PSO, and SA over 20 runs on six instances of different sizes. The 95-warehouse instance is taken as an example to illustrate the route construction of the optimal solution, as shown in Figure 5. In Figure 5, the light orange nodes represent warehouses in zone *A*, the light blue nodes represent warehouses in zone *B*, and the golden square denotes the port. Moreover, The results of the Wilcoxon signed-rank test are presented in Table 3. Lastly, the performance of IHSO and comparative algorithms is shown in Table 4 in two standard CVRP benchmark instances. Figure 6 illustrates the average convergence curves of the IHSO, HSO, ACO, GA, PSO, and SA over 20 runs on CVRPLIB benchmark instances.

As shown in Table 2, in six instances of different sizes, IHSO outperforms GA, PSO, SA, and HSO in the Best, Ave, and Std. In small-scale instances, IHSO outperforms ACO in the Best, Ave, and Std. In large-scale instances, ACO and IHSO show similar values in Std, but IHSO outperforms ACO in the Best and Ave. Taking all metrics into account, IHSO outperforms ACO. Moreover, the Best and Ave of IHSO are very close, which indicates that its solution quality possesses high stability. The results indicate that IHSO produces high-quality solutions while maintaining strong stability.

As shown in Table 3, the Wilcoxon signed-rank test was used to evaluate performance differences between IHSO and the compared algorithms. The results show statistically significant differences (*p* < 0.05) for all algorithms, which confirms the improvement achieved by IHSO.

Figure 4 shows that the solution of IHSO has faster convergence speed and higher quality. In six instances of different sizes, IHSO achieves a lower initial solution cost than GA, PSO, SA, ACO, and HSO, which shows that the greedy search algorithm can provide a better starting point and accelerate the convergence process. Figure 5 demonstrates that IHSO also outperforms the other algorithms in route construction. In summary, IHSO achieves well-structured routes, fast convergence, strong stability, and high solution quality in port cargo transportation planning.

As shown in Table 4, for the X-n308-k13 and X-n204-k19 CVRPLIB instances, IHSO outperforms the other compared algorithms in Best, Ave, and Std, demonstrating that the solutions obtained by IHSO are both high-quality and highly stable. For warehouse sizes of 204 and 308, IHSO reduces the optimal route length by 3.68% and 5.24% compared with ACO, respectively. As shown in Figure 6, IHSO achieves both the lowest path length and the fastest convergence among all compared algorithms. In addition, the greedy algorithms generate high-quality initial solutions, which accelerates the convergence speed of the algorithm.

## 5. Conclusions

To avoid the potential risks of centralized cargo storage at ports, this paper proposes a two-stage allocation–transportation framework based on IHSO. In the allocation stage, to avoid total loss of the same cargo type, a distance-based clustering method is employed to partition warehouses into two zones. Then, cargo is allocated to each warehouse according to the zone partitioning and warehouse capacity. In the transportation stage, this paper designs IHSO to plan the routes of port cargo transportation. IHSO integrates the greedy search algorithm, ACO, and adaptive swap/reversal operations with HSO, which enhances global search capability and significantly shortens the length of transportation routes. To evaluate the performance of IHSO, this paper compares IHSO with ACO, GA, PSO, HSO, and SA on instances of different scales. Experimental results demonstrate that IHSO shows superior solution quality compared with the other algorithms. Specifically, the experimental results on the self-generated instances demonstrate that IHSO reduces the optimal route length by up to 2.08% compared with the best-performing baseline ACO.

The experimental results on the CVRPLIB benchmarks demonstrate that IHSO reduces the optimal route length by up to 5.24% compared with the best-performing baseline ACO. Moreover, all improvements are statistically significant under the Wilcoxon signed-rank test (*p* < 0.05), which confirms that the advantage of IHSO over the baselines is systematic and reproducible rather than incidental.

However, this study still has some limitations. Currently, the research mainly focuses on optimizing the transportation length, while more complex and practical aspects still need to be fully considered, such as multi-objective dynamic scheduling, a stochastic disaster factor, ablation analysis of each module, and parameter-sensitivity analyses. Future research can further explore the application of IHSO in multi-objective dynamic scheduling [42,43,44,45], perform ablation experiments to analyze the effectiveness of each module, conduct a systematic parameter-sensitivity analysis to assess the influence of the key parameters, and introduce a disaster variable to quantitatively analyze the benefit of the two-zone dispersion relative to centralized storage.

## Figures and Tables

**Table 1:** Parameters of the improved holistic swarm optimization algorithm.

Algorithm	Parameter
IHSO	$\Phi_{\mathrm{final}}$ = 0.1, $\Phi_{\mathrm{init}}$ = 0.6, ζ = 0.99, T0 = 5000, α = 1, β = 1, ρ = 0.5, Q = 100

**Table 2:** Performance comparison of different algorithms in six instances.

Number of Warehouses	Performance	IHSO	HSO	ACO	GA	PSO	SA
95	Best	**28,322.77**	34,380.44	28,471.85	29,136.99	32,499.31	34,351.04
	Ave	**28,537.01**	34,933.70	28,779.01	29,842.45	33,954.82	35,124.39
	Std	**77.27**	360.81	116.18	385.36	675.79	370.60
185	Best	**53,030.02**	67,930.58	53,503.88	56,461.58	63,839.88	65,927.75
	Ave	**53,327.65**	69,398.41	53,963.31	57,775.74	65,797.37	67,453.79
	Std	**130.05**	518.40	184.68	577.91	777.26	566.38
271	Best	**74,966.17**	99,734.63	76,560.10	82,763.48	93,664.76	96,857.33
	Ave	**75,375.77**	101,462.63	77,245.30	84,255.01	95,824.02	98,027.91
	Std	**200.83**	571.03	209.94	919.93	1245.07	708.70
374	Best	**97,715.29**	138,235.40	99,534.36	114,363.82	128,943.86	131,362.71
	Ave	**98,225.82**	139,424.44	100,109.98	116,774.14	132,015.47	133,570.62
	Std	287.39	548.02	**269.65**	1066.87	1626.05	1154.13
463	Best	**124,504.80**	174,895.13	126,841.15	146,538.49	163,583.20	165,384.83
	Ave	**125,314.46**	176,075.12	127,428.94	148,344.69	167,431.96	168,096.62
	Std	374.18	460.60	**276.23**	814.31	1994.14	1396.01
559	Best	**155,989.07**	211,870.63	158,330.22	180,987.21	201,605.49	201,510.41
	Ave	**156,617.60**	213,549.13	158,997.39	182,872.47	204,976.35	204,390.34
	Std	354.91	568.93	**332.67**	1229.01	1558.49	1477.73

Note: Bold values indicate the best objective function results among all compared algorithms.

**Table 3:** Wilcoxon signed-rank test (*p*-value) in six instances.

Number of Warehouses	HSO	ACO	GA	PSO	SA
95	9.5 × 10^−7^	1.91 × 10^−6^	9.5 × 10^−7^	9.5 × 10^−7^	9.5 × 10^−7^
185	9.5 × 10^−7^	9.5 × 10^−7^	9.5 × 10^−7^	9.5 × 10^−7^	9.5 × 10^−7^
271	9.5 × 10^−7^	9.5 × 10^−7^	9.5 × 10^−7^	9.5 × 10^−7^	9.5 × 10^−7^
374	9.5 × 10^−7^	9.5 × 10^−7^	9.5 × 10^−7^	9.5 × 10^−7^	9.5 × 10^−7^
463	9.5 × 10^−7^	9.5 × 10^−7^	9.5 × 10^−7^	9.5 × 10^−7^	9.5 × 10^−7^
559	9.5 × 10^−7^	9.5 × 10^−7^	9.5 × 10^−7^	9.5 × 10^−7^	9.5 × 10^−7^

**Table 4:** Performance comparison of different algorithms in two CVRPLIB benchmark instances.

Instance	Performance	IHSO	HSO	ACO	GA	PSO	SA
X-n204-k19	Best	**21,540.00**	80,310.00	22,362.00	51,742.00	67,933.00	64,339.00
	Ave	**22,326.90**	83,692.00	23,393.25	55,501.15	72,702.60	68,667.35
	Std	**326.13**	1431.18	462.32	1461.83	2013.80	1688.80
X-n308-k13	Best	**33,749.00**	143,624.00	35,616.00	96,356.00	122,556.00	106,915.00
	Ave	**34,660.95**	149,264.40	36,360.85	99,997.70	129,901.90	111,123.25
	Std	**337.29**	1679.73	427.54	2548.96	3887.69	2518.34

Note: Bold values indicate the best objective function results among all compared algorithms.

## Data Availability

The data that support the findings of this study are available from the corresponding authors upon request.
